# Supplementary material for: Are carbon emissions trading and green financial instruments synergistic? -Comprehensive quantitative research based on content analysis
Source: PLoS One. 2024 Mar 7;19(3):e0298601. doi: 10.1371/journal.pone.0298601 (PMC10919601; doi:10.1371/journal.pone.0298601)
Supplement: S1 Data — (ZIP) [file pone.0298601.s001.zip › Data/地级市Z_F工作报告.zip!/║╙─╧/╞╜╢Ñ╔╜╩╨/└┤╫╘╣1⁄2╓┌║┼í╛┬φ┐╦╩2╛▌═°í┐_2018.docx]

各位代表:

现在，我代表市人民政府，向大会报告工作，请予审议，并请各位政协委员和其他列席人士提出意见。

一、2017年及本届政府工作回顾

2017年，面对复杂的经济发展环境和繁重的改革发展稳定任务，在市委的坚强领导下，全市上下以习近平新时代中国特色社会主义思想为指导，全面落实党中央、国务院和省委、省政府决策部署，主动适应经济发展新常态，坚持稳中求进工作总基调，以新发展理念为引领，以推进供给侧结构性改革为主线，着力发挥优势打好“四张牌”，坚决打好四大攻坚战，经济社会发展稳中有进、稳中向好，主要经济指标增速提升、位次前移，呈现出多年未有的好局面。初步核算，全市生产总值增长8.1%，由2016年全省第17位提升到第11位；一般公共预算收入增长11.3%，由第15位提升到第9位；固定资产投资增长12.3%，由第17位提升到第1位；规模以上工业增加值增长8.3%，由第15位提升到第8位；社会消费品零售总额增长12%，由第14位提升到第9位；城镇居民人均可支配收入增长9.3%，由第18位提升到第2位；农村居民人均可支配收入增长8.7%，由第14位提升到第12位。一年来，我们主要做了以下工作：

（一）抓关键、稳运行，推动经济平稳增长。持续深化企业服务，充分发挥政府与重点企业联席会议作用，切实为企业解难题、办实事，盘活了一批生产经营暂时遇到困难的企业。着力扩大有效投资，开展重点项目集中开工，加强重点工作督导，4万吨差异化尼龙66工业丝、万达广场等400多个项目开工建设或建成投用；争取中央预算内投资项目55个，总投资123亿元的国家储备林项目获批。推广运用政府和社会资本合作模式，PPP项目入库数、落地数分别居全省第1位、第3位；拓宽企业融资渠道，资本市场直接融资148.8亿元，银行业金融机构贷款新增137.1亿元；补充耕地占补平衡指标1.2万亩，拆旧复耕3.3万亩。出台转变政府职能优化营商环境实施意见，成立营商环境监督局，努力打造良好营商环境。

（二）聚合力、强举措，全力打好四大攻坚战。把四大攻坚战摆到十分突出的位置，统筹协调、加压推进，取得良好成效。脱贫攻坚扎实推进。按照“六个一”“三个零差错”“三个显著提升”要求，开展百日会战，实施十大专项行动，投入财政扶贫资金8.5亿元，5万人实现脱贫。“千头线”产业扶贫、健康扶贫创新做法得到省委、省政府肯定，全省文化扶贫、教育扶贫现场会在我市召开。国企改革攻坚如期完成任务。4家省属企业共分离移交供水12.6万户、供电12.2万户、供热3.2万户、物业服务12万户、退休人员社会化管理7.4万人、医疗保险生育保险属地管理21.5万人，被省委、省政府授予国企改革攻坚突出贡献单位。污染防治攻坚成效明显。强力推进十大攻坚行动，整治取缔“散乱污”企业8200多家，PM10和PM2.5平均浓度持续下降，优良天数显著增加，是年度考核9个优秀省辖市之一。集中式饮用水水源地水质达标率100%，地表水责任断面水质达标率96.4％、居全省第3位。转型发展攻坚进展顺利。出台“1+5”工作方案，明确12个重点攻坚产业，设立首批50亿元产业升级引导基金，产业项目投资增长12.1%，由全省第16位提升到第5位。高新技术产业集聚区、新华区特色商业区和宝丰县特色商业区晋级二星。

（三）促改革、扩开放，不断增强发展活力。认真落实“去降补”任务，关闭252万吨煤炭产能和30万千瓦火电机组，全部取缔地条钢；房地产去化周期降至12个月；统筹推进40项降成本措施，减轻企业负担7.15亿元。城市公立医院、盐业体制、城市综合执法体制等改革顺利推进，六大投融资公司全部实现规范化运营。推动河南城建学院建设大学科技园、平顶山学院建设创新创业园区，抗艾滋病一类新药阿兹夫定获得国家专利金奖，100多项先进科技成果有效转化，新增15家国家高新技术企业、6家省级重点实验室、10家省级工程技术研究中心和1家院士工作站。坚持把开放招商作为“一号工程”，大力开展专题招商、以商招商和产业链招商，实际利用外商投资增长7%，居全省第6位；进出口总值增长22%，居全省第5位。

（四）补短板、强功能，推动城市提质发展。按照“七个提质”要求，出台百城建设提质工程攻坚方案，启动全域规划编制，开展城市总体规划修编，完成城市地下空间利用、全域供热管网等规划，被列入全国新一轮土地利用总体规划编制试点市。谋划实施百城建设提质工程重点项目392个，完成投资114亿元，新新路北延、未来路北延、新南环路东延等工程完工；大西环、平叶快速通道开工建设；新增供热面积303万平方米、管网32公里，集中供热普及率达60.9%。问题楼盘化解、拆违拆迁、打通断头路、违法违规广告牌治理等工作成效明显。湛河支沟治理截污工作全部完成。汝州、舞钢、郏县百城建设提质工程试点工作取得积极进展。

（五）抓产业、固基础，扎实做好“三农”工作。大力发展现代农业，被确定为首批国家农业可持续发展试验示范区和农业绿色发展试点先行区。新增有效灌溉面积9.7万亩，新建高标准粮田21.8万亩，粮食总产206.2万吨。上海授予我市优质农产品供沪外延保障基地，完成农产品供应30多亿元。市烟草科技园建成，烟草业转型升级走在全省前列。新造林39.8万亩，建设生态廊道730公里，全省林业生态建设现场会在我市召开。解决131个贫困村15.5万人饮水安全问题。建立四级河长制，开展清河行动，完成首轮巡河。农村土地承包经营权确权登记颁证、宅基地和集体建设用地确权登记发证工作基本完成。创建全国文明村镇11个、省级文明村镇13个，农村人居环境明显改善。

（六）惠民生、增福祉，促进社会和谐稳定。财政民生支出增长16.6%，重点民生实事工作受到省政府表彰。落实困难群众大病补充医疗保险，建档立卡贫困人口参保率100%，特困人员救助供养对象、城乡低保对象、困境儿童参保率98%。“全面改薄”校舍类学校开工率和竣工率均居全省第1位，仪器设备类学校全部通过验收。市第二人民医院病房综合楼、精神卫生中心主体完工。46家城市公立医院全部取消药品加成，家庭医生签约率62.2%，实现对贫困人口的全覆盖。新开工保障性住房6534套、完成116.6%，货币化安置2518套、完成108.4%。加强食品药品安全监管，在全省综合考核中居第2位。集中开展安全生产大检查和专项整治，保持全省安全生产优秀位次。依法打击处置非法集资，推进立体化社会治安防控体系建设，有效化解信访案件，全面打赢党的十九大安保维稳攻坚战，社会大局保持和谐稳定。

各位代表！

本届政府任期的四年，我们砥砺奋进、拼搏实干，经济社会发展取得了新成绩，在决胜全面建成小康社会进程中迈出了坚实步伐。

四年来，我们坚持发展第一要务，综合实力稳步增强。生产总值突破2000亿元，年均增长7.4%；固定资产投资累计完成6730.2亿元，年均增长12.1%；一般公共预算收入达到137.5亿元，成功跨越产业转型期下滑区间；金融机构人民币存贷款余额分别达到2542.1亿元和1726.5亿元，规模以上工业主营业务收入突破2700亿元，经济社会发展站上了更高的平台。

四年来，我们加快产业结构调整，转型发展迈出新步伐。现代生态循环农业创新实践走在全国全省前列。第三产业规模水平不断提升，占生产总值比重达到42.6%，对全市经济增长贡献率达到47.5%，分别提升11.3个和17.5个百分点。第二产业结构持续优化，非煤产业、装备制造业、高新技术产业比重分别提升10个、3.8个、4.1个百分点，尼龙新材料建成及在建产能突破300万吨，工业盐及盐化工产能突破600万吨，炼钢及钢材制品产能突破1100万吨，正在由中原煤仓向国际新材料产业基地转变。

四年来，我们深化改革开放创新，发展动能加快转换。 “放管服”改革扎实推进，精简各类权限1078项，非行政许可审批全面取消。商事制度改革成效明显，日均新登记企业数量是改革前的2.4倍，市场主体达17.7万户。户籍制度改革和居住证制度在全省率先实现“双落地”。中房公司、市自来水公司完成改制，平顶山日报传媒集团挂牌成立，5个县（市、区）完成农信社改制。10家企业在新三板挂牌。郑州海关平顶山办事处获批。开展系列重大招商活动，实际到位省外资金年均增长12.6%，进出口总额年均增长10.7%。国家高压电器产品质量监督检验中心、炼焦煤资源开发及综合利用国家重点实验室落户我市，国家特色蔬菜产业体系韭菜岗位科学家设在市农科院，建成平煤神马集团上海联合研发中心、平高集团清华大学联合研究中心、平顶山市健康食品协同创新中心，省级以上研发平台达到147家，成为全国科技进步先进市、国家知识产权试点市。

四年来，我们加强基础能力建设，发展优势不断增强。光伏和风力发电装机突破100万千瓦，热力管网突破300公里，天然气管网突破1000公里，有线宽带和4G网络覆盖所有行政村。南水北调中线工程建成通水，调水及生态补水3亿多立方米，战胜建市以来最严重旱灾。高速公路突破500公里，国省干线公路突破1700公里，新改建农村公路2200多公里，三洋铁路郏县段、孟平铁路复线建成投运，平宝、平郏、宝石快速通道建成通车，郑万铁路、沙河复航等项目加快建设，鲁山机场前期工作持续推进，现代大交通格局初步形成，正在向区域性综合交通枢纽挺进。

四年来，我们统筹区域协调发展，城乡面貌深刻变化。成功纳入中原城市群核心发展区和淮河生态经济带规划，创建为国家卫生城市和省文明城市。常住人口城镇化率52.4%，年均提高1.5个百分点。县城和小城镇建设取得新成效，率先在全省实现园林县城全覆盖。全面开展农村人居环境整治，建设达标村1410个、示范村593个。中心城区建成区面积突破100平方公里，白龟湖国家湿地公园通过验收，湛河综合治理取得显著成效，湖滨新城形象日益凸显，正在由矿城向宜居宜业城市转变。

四年来，我们坚持以人民为中心，人民生活持续改善。集中力量办好重点民生实事，财政民生累计支出841亿元，新增城镇就业45.6万人，新增农村劳动力转移就业22.5万人，22.4万农村贫困人口稳定脱贫。城镇和农村居民人均可支配收入分别达到2.96万元和1.22万元。居民基础养老金最低标准、基本医疗保险补助标准分别提高36.7%和40.6%。新建改扩建幼儿园229所、中小学360所，高考本科上线率实现8连增，平顶山学院被确定为省示范性应用技术类型本科院校，平顶山教育学院改制为职业技术学院。宝丰县被命名为国家级说唱文化生态保护实验区。成功举办市第九届运动会。建立分级诊疗新模式，公共医疗卫生服务体系更加健全。民生和社会事业发展步伐明显加快。

四年来，我们扎实推进政府系统全面从严治党，党的群众路线教育实践活动、“三严三实”专题教育、“两学一做”学习教育深入开展。加强法治政府建设，自觉接受人大监督、政协民主监督和社会监督，认真办理人大代表建议和政协委员提案。加强国防教育和国防后备力量及人民防空建设，支持军队改革发展，推进军民融合深度发展，驻平解放军、武警部队、预备役人员、民兵为地方发展作出新贡献，实现全国双拥模范城“五连冠”。妇女儿童、残疾人、红十字、慈善等事业健康发展。民族宗教、审计、统计、侨务、史志、档案、气象、地震等工作取得新成绩。

各位代表！

经过四年的发展，全市经济社会发展迈上了新台阶，进入了蓄势崛起的新阶段，成绩来之不易。我们深切感受到，这些成绩的取得，最根本在于有以习近平同志为核心的党中央的正确领导，有习近平新时代中国特色社会主义思想的科学指导，有习近平总书记视察指导河南时重要讲话精神的方向指引；得益于省委、省政府和市委的坚强领导，得益于市人大、市政协和社会各界的支持监督，得益于全市人民的团结奋斗。在此，我代表市人民政府，向全市广大干部群众，向各民主党派、人民团体和社会各界人士，向驻平部队和武警官兵，致以崇高敬意！向关心支持平顶山发展的海内外友好人士，表示诚挚感谢！

各位代表！

成绩属于过去，未来更需努力。我们清醒认识到，经济社会发展中仍面临不少困难和问题：一是经济增长动力不足，创新能力不强，民营经济发展相对滞后，企业特别是行业龙头企业数量偏少。二是产业结构矛盾依然突出，第一产业规模化和品牌化发展不够，第二产业结构调整任务艰巨，第三产业仍是转型发展的短板。三是风险隐患增多，企业风险、金融风险、政府债务风险和安全生产风险不容忽视，处置非法集资、化解问题楼盘仍需付出很大努力。四是民生领域仍有短板，脱贫攻坚和污染防治任务较重，城市基础设施建设欠账较多，就业、教育、医疗、养老等方面还不能很好满足人民群众的需要。五是政府职能转变还不到位，一些政府工作人员能力素质不适应新时代要求，一些领域不正之风和腐败问题不容忽视。对此，我们一定高度重视，勇于担当，认真解决，决不辜负全市人民的期盼！

二、今后五年的总体要求和目标任务

今后五年是决胜全面建成小康社会、开启全面建设社会主义现代化新征程的关键时期。当前，我国经济发展进入新时代，由高速增长转向高质量发展阶段。推动高质量发展，我们既面临不少压力和挑战，也具备诸多有利条件，全国全省经济持续稳中向好，党的十九大报告明确提出“支持资源型地区经济转型发展”，中央、省出台的一系列政策措施效应正在持续释放；我市各类资源相对富集，产业基础较好，市场主体素质不断提升，发展环境不断改善，干部作风大整顿更激发了全市干部新时代新担当新作为的强大正能量。只要我们转变理念、遵循规律，锲而不舍、攻坚克难，就一定能够开拓高质量发展新境界。

今后五年发展的总体要求是：全面贯彻落实党的十九大精神，以习近平新时代中国特色社会主义思想为指导，认真落实习近平总书记视察指导河南时的重要讲话精神，聚焦持续推进“四个着力”、发挥优势打好“四张牌”重大要求，坚持稳中求进工作总基调，坚持新发展理念，坚持高质量发展根本方向，紧扣社会主要矛盾变化要求，统筹推进“五位一体”总体布局和协调推进“四个全面”战略布局，以供给侧结构性改革为主线，统筹稳增长、促改革、调结构、惠民生、防风险，着力打好决胜全面建成小康社会“三大攻坚战”，着力推进“五大转型”，着力深化改革开放创新，着力实施乡村振兴战略，着力改善民生维护稳定，促进经济社会持续健康发展，决胜全面建成小康社会，综合实力高质量重返全省第一方阵，高起点开启新时代建设社会主义现代化新征程，为中原更加出彩贡献力量、增添重彩。

建议今后五年经济社会发展的主要目标是：通过全力晋位次争上游走前列，推动经济在高质量发展中保持平稳健康增长，转型发展取得显著成效，社会治理体系和治理能力现代化水平显著提高，到2020年全面建成小康社会，并乘势而上全面推进新时代社会主义现代化建设，持续提升平顶山在全省发展大局中的地位，更好满足人民对美好生活的向往。

——综合实力明显提升。经济发展的平衡性、包容性、可持续性显著提高，生产总值年均增长7.5%以上，常住人口城镇化率达到60%左右，城镇化质量明显提升，乡村振兴取得重要进展，社会文明程度显著提高。

——产业转型明显加快。工业“1+7”制造业产业体系日益完善，服务业实现扩量升级，现代农业加快发展，多元发展、多极支撑的现代产业格局基本形成，创建成为国家老工业基地和资源型城市产业转型升级示范区。

——生态环境明显改善。绿色低碳循环发展持续推进，能源资源开发利用效率进一步提高，单位生产总值能耗下降15%，主要污染物排放总量大幅减少，大气污染治理取得决定性成效，水环境质量根本改善，土壤环境质量逐步好转。

——社会事业明显进步。教育、文化、卫生、体育等基础设施和公共服务体系更加健全，多层次社会保障体系更加完善，基本公共服务均等化水平明显提高，科技创新体系全面构建，全民文化素养显著提升，鹰城文化更具魅力，创建为全国文明城市。

——民生福祉明显增进。全市现行标准下农村贫困人口全部脱贫，贫困县全部摘帽。城乡居民收入与地区生产总值同步增长，城乡居民收入差距进一步缩小，人民受教育程度和健康水平不断提升，就业比较充分，居民生活条件和生活质量全面改善。

——社会治理明显高效。社会主义核心价值观深入人心，民主法治建设取得重大进展，法治政府基本建成，司法公信力明显提高，法治意识普遍增强，彰显公平正义、共建共治共享的社会治理格局基本形成。

工作中要始终坚持以习近平新时代中国特色社会主义思想为指导，把握好以下原则：一是必须坚定不移坚持党对一切工作的领导。牢固树立“四个意识”，坚决做到“两个维护”，不折不扣贯彻落实党中央决策部署，以党的建设高质量推动经济发展高质量。二是必须坚定不移坚持以人民为中心。始终牢记全心全意为人民服务根本宗旨，把更好满足人民日益增长的美好生活需要作为发展的根本目的，让人民群众有更多获得感、幸福感、安全感。三是必须坚定不移推动高质量发展。深入贯彻新发展理念，积极推进产业、城市、生态、文化、社会“五大转型”，加快质量变革、效率变革、动力变革，努力走出一条资源型城市高质量转型发展新路。四是必须坚定不移深化改革开放创新。坚持全面深入地推进各项改革，发展更高层次开放型经济，大力实施创新驱动战略，让市场机制更为有效、微观主体更具活力、政府作用更好发挥。五是必须坚定不移推进晋位争先创优。咬定转型发展目标不动摇，保持争先晋位劲头不懈怠，持续转变工作作风，大力弘扬焦裕禄同志的“三股劲”，全力推动各项工作晋位次争上游走前列，切实推动我市综合实力高质量重返全省第一方阵。

三、2018年重点工作

根据市委经济工作会议要求，综合各方面因素，建议2018年全市经济社会发展主要预期目标为：生产总值增长7.5%以上，规模以上工业增加值增长7.5%以上，固定资产投资增长10%左右，社会消费品零售总额增长11%左右，进出口保持平稳增长，一般公共预算收入增长12%以上，税收占一般公共预算收入比重71%以上，居民人均可支配收入增速略高于经济增长，新增城镇就业5.78万人以上，城镇调查失业率和城镇登记失业率分别控制在5.5%和4.5%以内，常住人口城镇化率提高1.5个百分点以上，研究与试验发展经费占生产总值的比重进一步提高，空气优良天数219天以上，居民消费价格涨幅控制在3%以内，单位生产总值能耗降低6%，节能减排、环境保护等指标完成省下达任务。

今年以来，全市上下认真履职尽责、积极担当作为，经济社会发展延续了平稳健康、持续向好的态势。经济保持平稳增长。上半年，生产总值增长8％，高于全省0.2个百分点。1-8月份，固定资产投资增长11.2%，高于全省2.7个百分点，居全省第2位；规模以上工业增加值增长8.1%，高于全省0.6个百分点，居全省第4位；一般公共预算收入增长15.9%，高于全省0.7个百分点。三大攻坚扎实推进。深入开展脱贫百日攻坚提升行动，上半年脱贫攻坚考核居全省第8位，打了一个翻身仗。PM10、PM2.5平均浓度和优良天数三项指标实现“两降一增”，集中式饮用水水源地水质达标率保持100%。问题楼盘化解工作有序推进，僵尸企业处置工作提前完成，政府债务率控制在省定标准之内。发展新动能不断增强。出台政务服务“一次办妥”改革实施方案，公布实施390项“一次办妥”事项。成功举办尼龙新材料产业技术发展大会等活动，建立招商引资签约项目全程代办服务制，累计到位省外资金390.4亿元，实际吸收外资增长54.1%。入库国家科技型中小企业172家，专利授权量1602件、增长99.5%。人民生活进一步改善。财政民生支出增长20.5%，占一般公共预算支出比重74.7%。上半年，城镇和农村居民人均可支配收入分别增长8.7%和9.1%，均超过生产总值增速。完成拆违拆迁690万平方米，争取棚户区改造项目6.7万套，居全省第4位。村（社区）两委换届选举工作全部完成。安全生产形势持续稳定，扫黑除恶专项斗争深入开展，公众安全感持续提升，社会大局保持和谐稳定。总体看，今年以来，全市主要经济指标增速高于全省平均水平，各项工作扎实推进，为全年各项目标任务的完成奠定了坚实基础。我们将开拓创新、奋发进取，持续抓好以下十方面工作：

（一）坚决打好三大攻坚战，夯实全面建成小康社会基础。围绕年度攻坚任务，明确各方责任，强化政策保障，把各项工作做实做好，为全面建成小康社会提供前提条件。

打好防范化解重大风险攻坚战。坚持底线思维，抓好重点领域风险防范化解工作，坚决守住不发生区域性系统性风险底线。坚决防范化解金融风险。认真履行属地金融风险处置监管责任，对苗头性、倾向性问题早识别、早预警、早处置，堵住风险源头；加快公共信用信息平台建设，完善政务诚信、个人诚信等社会信用体系；持续打击非法集资、金融犯罪、网络传销等活动；稳妥有序推进问题楼盘化解工作。严格防控地方债务风险。加强政府债务审计，严格控制增量，稳妥化解存量，优化政府债务结构；合理安排政府债券，规范政府购买服务，坚决查处各类违规举债、担保等行为。稳妥化解企业风险。深入推进市场化“债转股”，引导企业强化风险意识、加强财务管控、优化债务结构，加快资产证券化，降低债务杠杆率。

打好精准脱贫攻坚战。认真贯彻精准扶贫、精准脱贫基本方略，制定打赢脱贫攻坚战三年行动实施方案，聚焦深度贫困村、特殊贫困群体，尽锐出战、精准施策，下足“绣花”功夫，全面提高脱贫质量，实现5万农村贫困人口稳定脱贫、叶县脱贫摘帽。落实重点任务。按照脱贫攻坚提质增效32条工作标准，统筹非贫困县、非贫困村脱贫攻坚工作，抓好产业扶贫、金融扶贫、健康扶贫、文化扶贫、教育扶贫、电商扶贫、交通扶贫、支持外出务工人员返乡下乡创业、易地扶贫搬迁、农村人居环境整治等重点工作，深入开展百企帮百村、万商扶万家等活动，做好驻村帮扶、定点帮扶、结对帮扶等工作。激发内生动力。注重扶贫与扶志扶智相结合、救急纾困与内生脱贫相结合，加强政策宣传和教育引导，加大就业技能培训力度，建立正向激励机制，实现可持续稳定脱贫。加强贫困村基层基础建设，提升扶贫干部带领贫困群众脱贫致富的能力水平。加强督导考核。实行最严格的督查巡查和考核评估, 定期通报情况，严格兑现奖惩。开展扶贫领域腐败和作风问题专项治理，确保真脱贫、脱真贫。

打好污染防治攻坚战。认真贯彻落实习近平生态文明思想，加快生态转型，促进经济发展与环境改善良性互动。深入推进大气污染防治。坚持精准施策、标本兼治，大力调整优化产业结构、能源结构、运输结构和用地结构，着力控制污染源头，狠抓末端排放治理，强化“六控”措施落实，健全绿色环保调度和差别化管理制度，深入开展工业企业绿色革命，彻底整治北部矿区污染，优先发展城市公共交通，稳步推进清洁取暖，确保完成省定空气质量目标任务。持续推进水污染防治。深入实施“水十条”，全面落实河长制湖长制，强化重点流域和河流治理，加强城市生态水系建设和饮用水源保护，确保集中式饮用水水源地水质达标率保持100%、出境断面水质综合达标率完成省定目标，市区建成区黑臭水体全部消除，各县（市）城区基本完成黑臭水体截污纳管工作。启动土壤污染防治。深入实施“土十条”，扎实开展全国第二次污染源普查，实施农用地土壤环境分类管理和建设用地准入管理，强化农村农业面源污染防治，开展土壤污染治理与修复，严格危险废弃物环境监管，加强生活污染源管控，努力守护一方净土。加大生态保护修复力度。完善生态文明建设考核机制，开展领导干部自然资源资产离任审计。推进生态保护红线、永久基本农田、城镇开发边界三条控制线划定实施，推动绿色矿山建设，加强地质灾害防治。加快森林、湿地、流域、农田、城市五大生态系统建设，开展大规模国土绿化行动，抓好廊道绿化、北部山区绿化、国家储备林建设等重点工程，建好绿屏障。加强湿地公园建设，确保白龟湖国家湿地公园通过复验。

（二）持续扩大有效需求，促进经济稳定增长。注重发展实体经济，持续释放有效需求，推动经济发展行稳致远。

扩大有效投资。健全重点项目建设推进机制，持续开展重大项目集中开工活动，组织实施425个重点项目，完成投资880亿元以上。加强项目谋划，力争更多项目列入国家、省计划盘子。完善落实促进民间投资支持政策，有序规范推进PPP项目实施。

培育消费热点。把握消费升级趋势，充分挖掘健康、养老、教育、体育、文化、旅游等消费领域潜力，鼓励和引导节能、环保、低碳等绿色产品消费，扩大高品质产品和服务供给。推动医养融合化发展、居家社区养老服务社会化发展，加快实施康养家政服务扩容提质工程，推进一批养老中心建设试点。持续开展消费专项整治行动，依法惩处各类侵害消费者权益的行为。

服务好实体经济发展。深化企业服务，持续实施“百千万”助力企业成长计划，深入开展产销、银企、用工、产学研对接，切实为企业排忧解难。持续开展“退城进园”，推动企业实现装备更新、产品换代、产业提升。抓好降成本政策落实，实施好国家新的减税降费政策，降低企业成本。深化城镇规划区内建设用地、农村耕地、农村集体建设用地改革，保障项目用地需求。

激发各类市场主体活力。大力支持平煤神马集团、平高集团、舞钢公司等国有企业发展，推动国有资本优化资源配置、改善供给结构、延伸产业链条，形成产业集群。全面落实支持非公有制经济发展的政策措施，积极构建亲清新型政商关系，大张旗鼓支持民营企业发展。大力发展混合所有制经济，推动国有企业和民营企业实现优势互补、合作共赢。弘扬企业家精神，提振企业家信心，营造尊重、鼓励、保护企业家干事创业的社会氛围。

（三）深化供给侧结构性改革，提高发展质量效益。深入落实“破、立、降”要求，持续打好产业结构优化升级牌，推动经济高质量发展。

持续优化工业结构。严格执行环保、能耗、质量、安全等标准，全面完成煤炭等行业化解过剩产能任务。建立淘汰落后产能“清零行动”长效机制，巩固“僵尸企业”处置成果。按照“新、链、群、台”要求，高质量推进中国尼龙城建设，大力发展尼龙新材料和盐化工及PC、新能源储能、高端装备制造、特钢不锈钢、煤焦油深加工、碳新材料、健康食品等“1+7”制造业产业，重点抓好年产60万吨己内酰胺——聚合——纺丝一体化、40万吨聚碳酸酯、20万吨己内酰胺、西气东输平顶山盐穴储气库等重大项目建设，高标准规划建设石墨焙烧及石墨化产业园，支持平高集团电力储能和平煤神马集团高端储能产业发展。积极培育新医药产业，加大生物制药、医药中间体等研发力度，推动阿兹夫定、培南类抗生素等新特药产业化。

加快服务业发展。坚持市场需求引领、重点产业带动、服务能力提升，推进服务业扩量升级。树立全域旅游理念，加强旅游基础设施建设，抓好尧山文化旅游综合体、香山古镇旅游综合体、鲁山花瓷小镇等项目建设，支持汝州、舞钢、鲁山创建国家全域旅游示范区，支持宝丰创建省旅游标准化示范区，推进旅游与文化、城镇、工业、农业、生态融合，打造有影响力拳头产品和精品旅游线路。以冷链物流、快递物流、电商物流为重点，加强与中民投集团合作，积极引进知名物流企业，加快推进现代物流产业集聚区、泰顺达电商仓储物流园等项目建设，推进物流业转型发展。加快发展金融业，积极引进各类金融机构，推动地方金融机构完善现代金融企业制度，巩固提升农信社改制成果。

深化转型发展攻坚。深入实施12个重点产业转型发展专项方案，加快传统产业绿色改造、智能改造、技术改造，突出抓好结构改革、技术创新、企业成长和集群培育等重点工作，加快培育一批高技术、高成长、高税利产业。深入推进质量强市建设，继续实施增品种、提品质、创品牌专项行动，大力推进精益制造、品牌制造，增强供给产品适应市场需求的能力。继续实施标准化引领工程，引导企业瞄准国际国内先进水平，开展对标达标专项行动，以标准提升引领和倒逼质量变革。

（四）深入实施创新驱动发展战略，培育经济发展新动能。坚持把创新作为引领发展的第一动力，持续打好创新驱动发展牌，不断增强经济创新力和竞争力。

抓好“四个一批”。突出发展一批创新引领型企业、培育一批创新引领型人才、建设一批创新引领型平台、引进一批创新引领型机构，实施高新技术企业“双倍增”行动，推进大中型工业企业省级研发机构全覆盖，抓好大学科技园、众创空间等创新创业孵化载体建设，支持河南城建学院、市农科院建设院士工作站，培育高新技术企业20家，建设省级以上创新平台20家以上。

加大技术研发力度。瞄准产业转型升级的核心技术、关键技术、前沿技术，在高压电气、电子信息、新医药、新材料、智慧能源等领域，集中实施一批重大科技专项，力争在一些关键领域和环节实现突破。支持平煤神马集团与东华大学联合建设尼龙新材料研究院，推动河南城建学院与四川大学联合建设研发中心，提升聚酰胺中间体和高压开关两个省级重点实验室的规模层次。

深化科技体制改革。加强科技经费支出改革，推行科技创新券制度，优化科技基础资源配置。探索建立科技型中小企业信用贷款风险补贴制度，开展科技贷、科技保、知识产权质押贷款等业务。用好郑洛新国家自主创新示范区政策，推动国家创新型城市、国家创新驱动示范市、国家知识产权示范市创建，把高新区打造成科技体制改革先行先试示范区。

（五）强化基础能力建设，增创经济发展新优势。坚持突出重点、弥补短板，持续打好基础能力建设牌，蓄积发展后劲。

增强重大基础设施支撑能力。加快推进郑万铁路、沙河复航、大西环、大南环、北环路升级及西延、平叶和平鲁快速通道等项目建设，开工建设干线公路100公里，做好鲁山机场、许南公路东移、平宝路改线、绕城高速以及林唐、焦平高速平顶山段等项目前期工作。加强重点电网、天然气、热力和供水管网建设，开工、投产变电容量1762兆伏安、输电线路692公里，新增供热面积125万平方米，新建天然气管网50公里。编制全域循环生态水系规划，抓好北汝河综合治理、昭平台水库加固扩容等项目，推进城市应急引水、北部山区国储林引水等八大工程建设。

完善提升科学发展载体。着力抓好集群培育、节约集约、企业改造、产城融合等工作，推动产业集聚区提质转型发展。加快特色培育和功能提升，推动服务业“两区”提速增效发展。以推动新兴产业集群发展为重点，实施服务业专业园区建设行动，培育2家省级服务业专业园区。

强化人力资源支撑。树立人才资源是第一资源理念，加快推进人才强市战略，实施“鹰城英才计划”，制定强有力的人才引进办法，引进和培育一批高层次人才和创新团队。持续实施全民技能振兴和职教攻坚工程，完善技能培训与产业发展对接机制，全面提高劳动者技能素质。健全人才激励机制和人才服务保障体系，优化人才发展环境，让各类人才各得其所、大展所长。

（六）加快推进新型城镇化，推动城乡区域协调发展。坚持以人的城镇化为核心，持续打好新型城镇化牌，全面提高城镇化发展质量。

优化城镇布局。加强高铁片区、湛南新城等九大片区开发建设，有序疏解老城区功能，强化市区东部基础设施配套，进一步完善城乡一体化示范区功能，加快市中医院新城区分院、市第一人民医院新院区工程进度，推进四馆一中心、平煤神马集团研发中心、市委党校规划建设，提高中心城区综合承载能力。推动宝丰、叶县、鲁山、石龙区与中心城区协同联动发展，增强汝州、舞钢、郏县支撑地区发展能力。加快小城镇体制创新，科学推进特色小镇和小城镇布局建设。

深入推进百城建设提质工程。坚持规划引领，推进“多规合一”，编制全市国土空间规划，加快新一轮城市总体规划修编、全域规划和总体城市设计，高标准编制北部山区生态修复、通风廊道、街景整治等专项规划。开展生态修复和城市修补，推进海绵城市建设，抓好湛河支沟治理。加强基础设施和公共服务配套建设，断头路项目全部开工、21条建成通车，完成9条道路绿化改造、6个公园广场改造、15个游园提档升级、18座立交桥立体绿化，推进矿山公园、植物园、月季园、生态园建设。

强力推进“四城联创”。突出市容卫生治脏、交通秩序治乱、生态环境治污、公共服务治差，抓好市场摊点整治、老旧小区改造、“双违”拆迁、路口优化改造等重点工作，推进智慧城市建设，开展城市清洁、交通文明畅通和市民思想道德素质提升行动，着力提升城市品质，确保国家卫生城市、国家园林城市、国家森林城市复审顺利通过，全国文明城市创建取得扎实成效。

加快农业转移人口市民化。全面实行网上申请办理居住证，将住房保障、农民进城购房等纳入居住证基本公共服务范围。完善“人地、人钱”挂钩政策和细则，落实房屋租购同权，引导农业转移人口落户城镇。

（七）全面深化改革开放，激发经济发展动力活力。以纪念改革开放40周年为契机，推动改革开放取得新突破。

深化重点领域改革。深化国企改革，持续做好省属企业剥离办社会职能后续工作，支持推进14家驻平央企“三供一业”分离移交工作；积极推进企业产权结构、组织结构、治理结构改革，推动建立现代企业制度；加强国有资产监管，加快由管企业为主向管资本转变。深化“放管服”改革，纵深推进“多证合一”，全面推行市场准入负面清单制度，加快推行企业登记全程电子化和电子营业执照；深入推进“互联网+政务服务”，全面推进行政权力事项在线办理，打造“一次办妥”政务服务品牌。深化投融资体制改革，实行新版投资项目审批流程，进一步简化和规范投资项目审批事项，推进财政涉企资金基金化改革，加快投融资公司转型步伐。启动新一轮机构改革，持续推进高新区管理体制改革，深化行政执法、医药卫生、教育、盐业等体制改革。

持续扩大对外开放。全力抓好招商引资。树立“把老乡当老外、把民资当外资”的理念，支持本土企业扩大投资、新上项目，鼓励在外创业者带项目回乡创业发展。依托现有企业，完善配套服务设施，降低要素价格，瞄准重点区域，对接重点客商，实施驻地招商，持续开展以商招商、产业链招商和集群招商，探索股权招商、并购招商等新途径。大力发展对外贸易。巩固神马、舞钢、平高、平棉等出口品牌，培育平煤机、隆鑫机车等品牌，持续推动出口基地建设，扩大外贸出口份额。加强与阿里国际站等知名跨境电商平台的合作，加快跨境电子商务发展。支持能源、化工、装备制造等行业的优势企业走出去开拓国际市场。着力优化开放环境。加快海关机构建设，健全招商引资信息服务平台功能和外商投诉权益保护机制，切实保护内外资企业合法权益。深入落实优化营商环境实施意见，完善招商引资签约项目全程代办服务制度，营造稳定公平透明的营商环境。

（八）着力实施乡村振兴战略，推进农业农村现代化。科学编制实施乡村振兴战略规划，加快国家农业“两区”建设，推动农业提质增效、农村文明进步、农民增收致富。

大力发展现代农业。突出规模经营、特色经营，持续实施“循环农业+品牌农业+协同农业”发展战略，努力增加市场需要的高质量、绿色、无公害农副产品。认真落实粮食安全市长责任制，构建粮食安全长效机制。加强高标准粮田建设和中低产田改造，新增有效灌溉面积6万亩、农机总动力5万千瓦。大力发展现代生态循环农业，持续推进绿色种植和生态养殖行动计划，加快推进与泰国正大集团和中民投集团的合作，培育壮大“种养加”八大农业产业化联合体。规划建设健康食品产业园，发展都市生态农业、休闲观光农业和“互联网+农业”，推进农村一二三产业融合发展。实施农业品牌化战略，开展“三品一标”和“鹰城名优”农业品牌创建，抓好优质农产品供沪外延保障基地建设。加快农业科技创新，推进市农科院搬迁，规划建设韭菜科技田园综合体，打造绿色高效韭菜科研生产基地。强化农产品质量安全监管，推进农产品质量安全市创建。

全面深化农村改革。巩固农村土地承包经营权确权登记颁证成果，探索宅基地“三权”分置改革，落实第二轮土地承包到期后再延长30年政策，完成粮食生产功能区和重要农产品生产保护区划定工作。稳步推进农村房屋不动产登记，健全农村产权交易平台，抓好农村集体产权制度改革，大力发展农村集体经济。深化农村金融改革，支持金融网点和业务下沉，强化农业保险保障作用。深化集体林权、国有林场、供销社等改革，激发农业农村发展活力。

发挥农民主体作用。创新生产经营机制、社会服务体系、产业组织模式、乡村管理方式，提高农民组织化程度。大力培育新型农业经营主体、培养新型职业农民，发展多种形式适度规模经营。深入开展“回归工程”，支持外出务工人员返乡下乡创业，发展乡村旅游、农村电商等新业态。

加快美丽宜居乡村建设。围绕环境美、田园美、村庄美、庭院美，落实农村人居环境整治三年行动方案，实施“千村整治、百村示范”，加快垃圾、污水处理和村容村貌美化，推进农村能源革命、厕所革命，完善农村基础设施和公共服务。强化农村基层组织建设，打造懂农业、爱农村、爱农民的三农工作队伍。加强农村诚信建设，推动农村移风易俗。完善农村矛盾调解和便民服务机制，深度推广“四议两公开”，打造自治、法治、德治相结合的乡村治理体系。

（九）切实保障改善民生，促进社会和谐稳定。坚持以人民为中心，加大民生投入，让人民群众有更多获得感、幸福感、安全感。

扎实办好民生实事。一是继续提高城乡低保、基础养老金、特困人员生活费标准，巩固完善实施困难残疾人生活补贴和重度残疾人护理补贴制度。二是做好重点群体就业工作，新增城镇就业5.78万人以上。三是扎实推进大气污染防治攻坚，优良天数达到219天以上。四是推进“厕所革命”，县城及以上城市建成区、乡镇建成区、旅游景区新建改建公厕335座以上，中心城区建成区公厕全部达到二类及以上标准。五是继续免费开展预防出生缺陷产前筛查和新生儿疾病筛查，完成5.76万适龄妇女“两癌”免费筛查。六是完成全部贫困县和深度贫困村电网改造升级。七是抓好农村垃圾治理和畜禽养殖废弃物无害化处理，完成省定2个县（市、区）农村垃圾治理的达标验收。八是实施农村饮水安全巩固提升工程，解决深度贫困地区和2018年计划退出贫困县21个村的饮水安全问题。九是推进“四好农村路”建设，新建和改造农村公路330公里以上。十是实施“雪亮工程”和加强法律援助，实现重点部位、重点路段、复杂场所视频监控联网应用全覆盖，继续推进审判阶段刑事法律援助全覆盖。

着力扩大社会就业。落实就业创业政策，完善公共就业创业服务体系，促进高校毕业生、城镇困难人员、退役军人等重点群体多渠道就业创业。持续推动农村劳动力转移就业，加大对残疾人等就业困难人员援助力度，妥善解决化解过剩产能和处置“僵尸企业”过程中下岗分流职工再就业问题。依法治理拖欠农民工工资问题，维护劳动者合法权益。

健全社会保障体系。进一步健全收入分配机制，适当调整社会最低工资标准。实施全民参保计划，建成覆盖城乡居民的社会保障体系。积极参与养老保险全国统筹，全面完成机关事业单位养老保险改革。完善城乡居民基本医疗保险、大病保险、困难群众大病补充医疗保险和医疗救助制度，提高农村贫困人口门诊慢性病和门诊重特大疾病保障水平。全面推行以按病种付费为主的多元复合式医保支付方式，城乡居民基本医保财政补助提高到每人每年490元。适度提高优抚补助标准，健全临时救助制度。加强残疾康复服务。健全农村留守儿童、妇女、老年人关爱服务体系。严格落实抚恤优待政策。完善住房供给和保障体系，持续推进棚户区改造，扩大住房公积金制度受益面。

优先发展教育事业。实施第三期学前教育行动计划，完成“全面改薄”和扩充城镇义务教育资源五年规划任务，落实好消除大班额、寄宿制学校建设规划，着力缓解中小学生负担重、择校热等突出问题。持续改善贫困地区义务教育学校办学条件，落实乡村教师激励政策，推动城乡义务教育一体化发展。推进市六中、四十四中、实验中学整体重建。推动高等教育内涵式发展，支持平顶山学院、河南城建学院建设高水平应用型大学。深化产教融合、校企合作，提升职业教育办学水平。继续实施特殊教育提升计划。支持和规范社会力量兴办教育。加强师德师风建设，健全学生资助制度，努力让每个孩子享有公平而有质量的教育。

推进健康鹰城建设。深入推进分级诊疗、家庭医生签约服务和医联体建设，加快市第二人民医院病房综合楼、市精神卫生中心、市传染病医院新病房楼、市中心血站新址等项目建设。实施基层医疗卫生服务能力提升工程，新建、改扩建一批县级医院、妇幼保健和疾病防控机构，加强基层乡村医疗卫生机构标准化建设。健全公立医院补偿机制，支持社会办医。提升计生服务管理水平。广泛开展全民健身，巩固全民健身示范城市创建成果。实施食品安全战略，开展药品生产质量安全提升行动和食药保平安专项行动，推进食品安全示范市创建，让人民吃得放心。

加快文化强市建设。加强社会主义精神文明建设，运用好新时代文明实践中心、平顶山好人园、劳模园等宣传阵地，培育和践行社会主义核心价值观。深入推进优秀传统文化传承和群众性精神文明创建，开展公民诚信守法、文明风尚提升行动，大力弘扬开放、包容、务实、创新的城市精神。加快基层综合性文化服务中心建设，提升文化惠民工程效能，持续开展群众文化活动，繁荣文艺创作。加强历史文化遗产保护。实施好文化产业发展三年行动计划，推动文化产业高质量发展。做好广播电视、新闻出版工作，营造良好舆论氛围。深入开展“扫黄打非”行动。加强网络建设治理，营造清朗网络空间。

推动社会治理创新。加强社区规范化建设。完善依法维权和矛盾纠纷多元化解机制，有效解决各类信访问题，及时预防和化解各类群体性事件。推进突发事件预警信息发布体系建设，提升防灾减灾救灾能力。强化安全生产责任，深化重点地区、重点领域、重点行业隐患排查和专项整治，确保实现“一个杜绝、三个持续下降”目标。积极开展平安创建，加快立体化信息化社会治安防控体系建设，强力推进扫黑除恶专项斗争，依法打击各类违法犯罪和暴力恐怖活动，确保社会大局和谐稳定。

加强国防教育、国防动员和国防后备力量建设。支持国防和军队改革，支持驻平解放军、武警部队、预备役部队、民兵和人民防空建设，做好创建全国双拥模范城“六连冠”工作。促进民族团结、宗教和睦。支持工会、共青团、妇联等人民团体更好发挥桥梁纽带作用。做好外事、侨务、对台工作。开展好第四次全国经济普查和第三次全国国土调查。加强史志、档案、气象、地震、邮政等工作。

（十）切实加强自身建设，提升政府治理能力。顺应人民群众新期待，适应实践发展新要求，加强政府改革创新，建设人民满意的高效服务型政府。

着力打造政治过硬的政府。始终把政治建设放在首位，高举习近平新时代中国特色社会主义思想伟大旗帜，牢固树立“四个意识”，坚定“四个自信”，践行“两个维护”，始终在政治立场、政治方向、政治原则、政治道路上同以习近平同志为核心的党中央保持高度一致。严守政治纪律，恪守政治规矩，坚决贯彻中央大政方针，坚决落实中央、省委、市委决策部署，始终做到政治坚定、对党忠诚。

着力打造依法行政的政府。牢固树立法治理念，全面推进依宪施政、依法行政，严格依法履行职责，严格规范公正文明执法，严格执行重大行政决策法定程序，着力建设法治政府。自觉接受人大法律监督、工作监督和政协民主监督，主动接受社会监督和舆论监督，认真听取各民主党派、工商联、无党派人士和人民团体意见，办理好人大代表建议和政协委员提案。全面深化政务公开，确保权力在阳光下运行。

着力打造敢于担当的政府。推进“两学一做”学习教育常态化制度化，开展“不忘初心、牢记使命”主题教育，提升专业能力，倡导专业精神，努力增强“八种本领”，不断提高执政水平。深入开展干部作风大整顿，完善激励约束、容错纠错机制，旗帜鲜明给积极干事者撑腰鼓劲。大兴学习之风、调查研究之风、干事创业之风，敢于担当、勇于碰硬，以攻城拔寨的拼劲、抓铁有痕的韧劲，推动各项工作落到实处、取得实效。

着力打造清正廉洁的政府。认真履行全面从严治党主体责任和“一岗双责”，严格落实中央八项规定及实施细则精神，驰而不息整治“四风”，特别是力戒形式主义、官僚主义。深化廉政风险防控，加强审计监督，严管财政资金使用、公共资源交易、国有资产管理等重点领域和关键环节，坚决查处各类腐败问题，推动干部清正、政府清廉、政治清明。

各位代表！

新思想引领新时代，新目标开启新征程。让我们更加紧密地团结在以习近平同志为核心的党中央周围，在省委、省政府和市委的坚强领导下，锐意进取、埋头苦干，为决胜全面建成小康社会、推动平顶山综合实力高质量重返全省第一方阵、助力中原更加出彩而努力奋斗！
